# Supplementary figures and images for: A vacuum-actuated soft robot inspired by Drosophila larvae to study kinetics of crawling behaviour
Source: PLoS One. 2023 Apr 5;18(4):e0283316. doi: 10.1371/journal.pone.0283316 (PMC10075400; doi:10.1371/journal.pone.0283316)

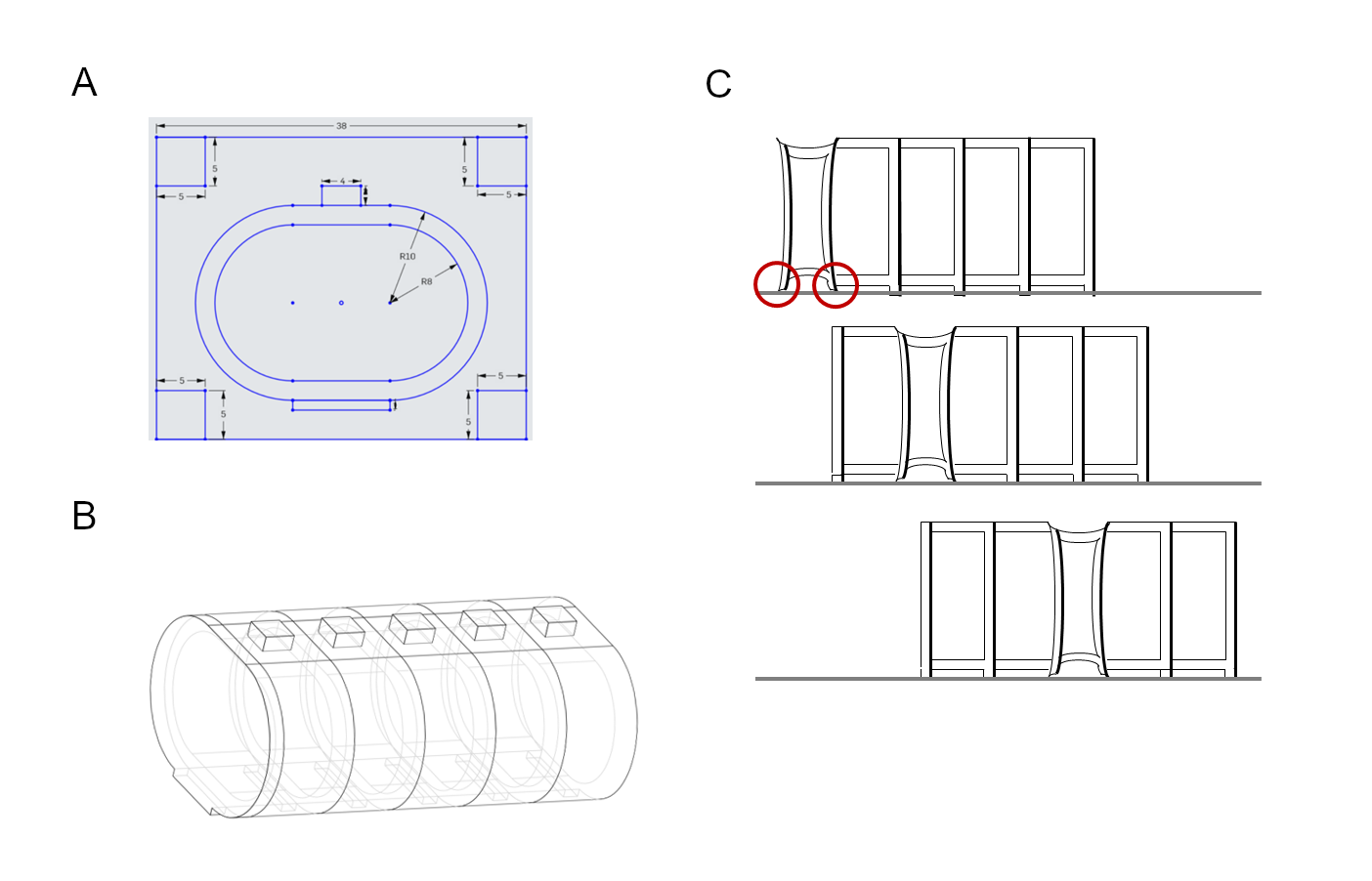

Supplement: S1 Fig — (A) Mould sketch for the soft structure. (B) The segmentally-repeated structure of the soft robot. (C) Locomotion scheme with segmental deformation. Red circles show the asymmetric friction between anterior and posterior segmental boundaries in body-substrate interaction. The left is anterior. Sheets of paper inserted in the soft robot are represented by thick lines. (TIF) [file pone.0283316.s001.tif]

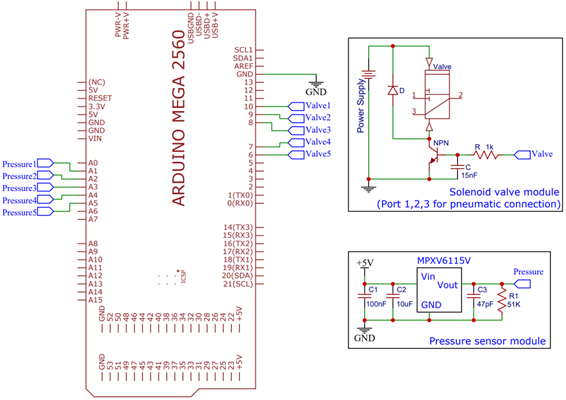

Supplement: S2 Fig — The left part represents the main connections for solenoid valves and pressure sensors. The right parts show the solenoid valve module (top) and pressure sensor module (bottom). (TIF) [file pone.0283316.s002.tif]

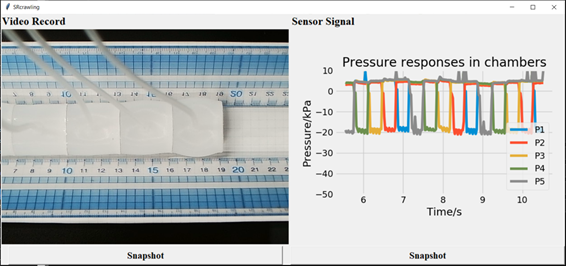

Supplement: S3 Fig — (TIF) [file pone.0283316.s003.tif]

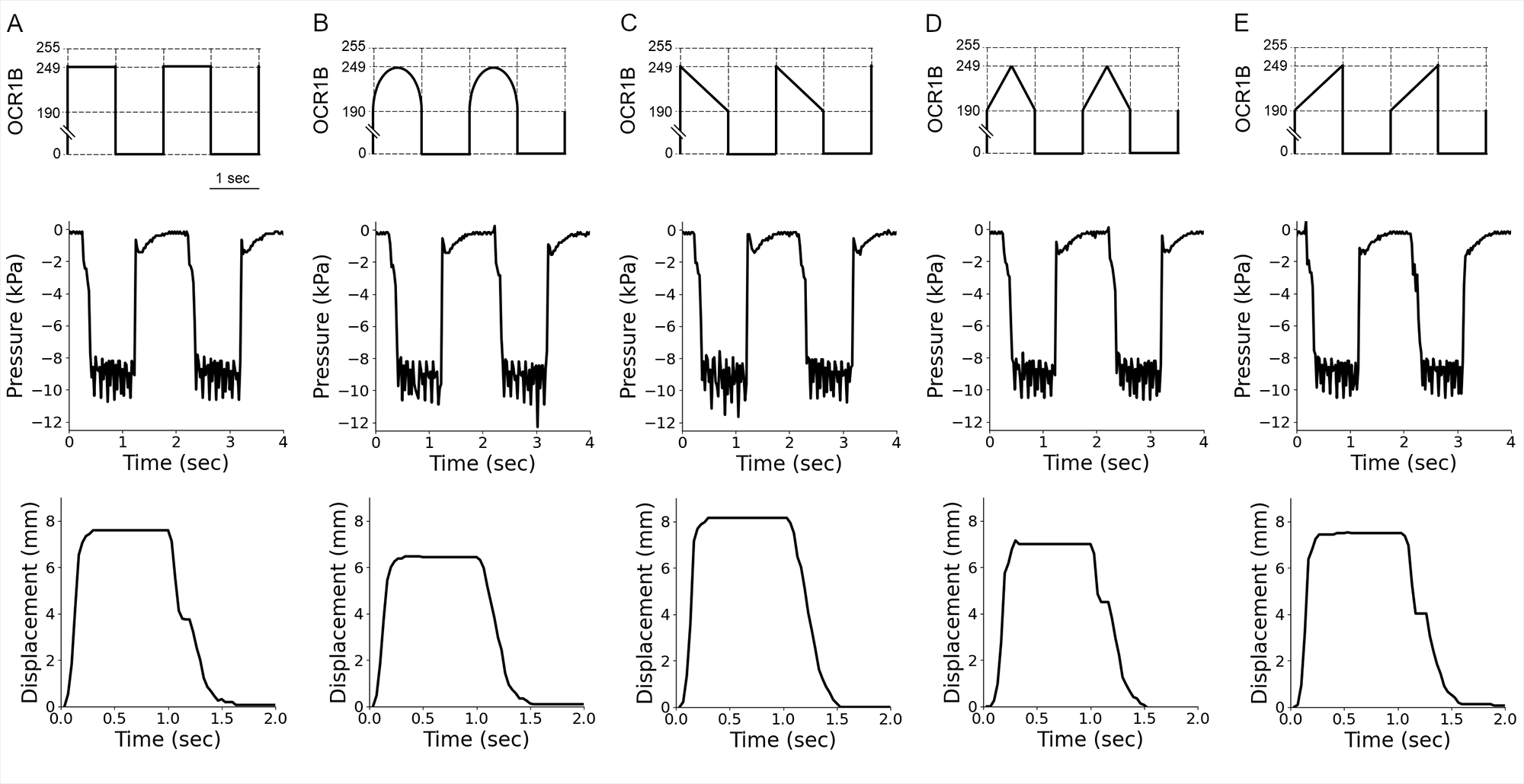

Supplement: S4 Fig — (A—E) The upper panels represent different control signals: square (A), sinusoid (B), and saw (C—E) waveforms. The middle panels show the pressure measured in the segmental chambers. The bottom panels represent the segment deformation induced by the control signal shown in the upper panels. (TIF) [file pone.0283316.s004.tif]
