# Supplementary material for: A vacuum-actuated soft robot inspired by Drosophila larvae to study kinetics of crawling behaviour
Source: PLoS One. 2023 Apr 5;18(4):e0283316. doi: 10.1371/journal.pone.0283316 (PMC10075400; doi:10.1371/journal.pone.0283316)
Supplement: Attachment — Submitted filename: PONE-D-22-28397_HB.pdf [file pone.0283316.s007.pdf]

PLOS ONE

A vacuum-actuated soft robot inspired by Drosophila larvae to study kinetics of crawling behaviour

--Manuscript Draft--

**Manuscript Number:**	PONE-D-22-28397
**Article Type:**	Research Article
**Full Title:**	A vacuum-actuated soft robot inspired by Drosophila larvae to study kinetics of crawling behaviour
**Short Title:**	A soft robot inspired by fly larvae
**Corresponding Author:**	Hiroshi Kohsaka, Ph.D.<br>The University of Electro-Communications: Denki Tsushin Daigaku<br>Chofu, Tokyo JAPAN
**Keywords:**	animal locomotion; crawling; fly larvae; soft robots; kinetics
**Abstract:**	Peristalsis, a motion generated by the propagation of muscular contraction along the body axis, is one of the most common locomotion patterns in limbless animals. While the kinematics of peristalsis has been examined intensively, its kinetics remains unclear, partially due to the lack of suitable physical models to simulate the locomotion patterns and inner drive in soft-bodied animals. Inspired by a soft-bodied animal, Drosophila larvae, we propose a vacuum-actuated soft robot mimicking its crawling behaviour. The soft structure, made of hyperelastic silicon rubber, was designed to imitate the larval segmental hydrostatic structure. Referring to a numerical simulation by the finite element method, the dynamical change in the vacuum pressure in each segment was controlled accordingly, and the soft robots could exhibit peristaltic locomotion. The soft robots successfully reproduced two previous experimental phenomena on fly larvae: crawling speed in backward crawling is slower than forward crawling, and limiting the duration of segmental motor bursts slows down larval peristaltic motion. Furthermore, our experimental results provided novel predictions for the functional roles of kinetic parameters, including intersegmental phase delay and contraction force, in peristaltic locomotion. These observations indicate that soft robots could serve to examine the kinetics of crawling behavior in soft-bodied animals.
**Order of Authors:**	Xiyang Sun, Ph.D.
	Akinao Nose, Ph.D.
	Hiroshi Kohsaka, Ph.D.
**Additional Information:**	
**Question**	**Response**
**Financial Disclosure**<br><br>Enter a financial disclosure statement that describes the sources of funding for the work included in this submission. Review the submission guidelines for detailed requirements. View published research articles from *PLOS ONE* for specific examples.<br><br>This statement is required for submission and **will appear in the published article** if the submission is accepted. Please make sure it is accurate.	This work was supported by MEXT/JSPS KAKENHI grants (17K19439, 19H04742, and 20H05048 to AN and 17K07042 and 20K06908 to HK). The funders did not play any role in the study design, data collection and analysis, decision to publish, or preparation of the manuscript.

Powered by Editorial Manager® and ProduXion Manager® from Aries Systems Corporation

**Unfunded studies**<br>Enter: *The author(s) received no specific funding for this work.*<br><br>**Funded studies**<br>Enter a statement with the following details:<br>• Initials of the authors who received each award<br>• Grant numbers awarded to each author<br>• The full name of each funder<br>• URL of each funder website<br>• Did the sponsors or funders play any role in the study design, data collection and analysis, decision to publish, or preparation of the manuscript?<br>• **NO** - Include this sentence at the end of your statement: *The funders had no role in study design, data collection and analysis, decision to publish, or preparation of the manuscript.*<br>• **YES** - Specify the role(s) played.<br><br>* typeset	
**Competing Interests**<br><br>Use the instructions below to enter a competing interest statement for this submission. On behalf of all authors, disclose any competing interests that could be perceived to bias this work—acknowledging all financial support and any other relevant financial or non-financial competing interests.<br><br>This statement is required for submission and **will appear in the published article** if the submission is accepted. Please make sure it is accurate and that any funding sources listed in your Funding Information later in the submission form are also declared in your Financial Disclosure statement.<br><br>View published research articles from *PLOS ONE* for specific examples.	The authors have declared that no competing interests exist.

**NO authors have competing interests**<br><br>Enter: *The authors have declared that no competing interests exist.*<br><br>**Authors with competing interests**<br><br>Enter competing interest details beginning with this statement:<br><br>*I have read the journal's policy and the authors of this manuscript have the following competing interests: [insert competing interests here]*<br><br>* typeset	
**Ethics Statement**<br><br>Enter an ethics statement for this submission. This statement is required if the study involved:<br><br>• Human participants<br>• Human specimens or tissue<br>• Vertebrate animals or cephalopods<br>• Vertebrate embryos or tissues<br>• Field research<br><br>Write "N/A" if the submission does not require an ethics statement.<br><br>General guidance is provided below. Consult the submission guidelines for detailed instructions. **Make sure that all information entered here is included in the Methods section of the manuscript.**	N/A

Powered by Editorial Manager® and ProduXion Manager® from Aries Systems Corporation

**Format for specific study types**<br><br>**Human Subject Research (involving human participants and/or tissue)**<br>• Give the name of the institutional review board or ethics committee that approved the study<br>• Include the approval number and/or a statement indicating approval of this research<br>• Indicate the form of consent obtained (written/oral) or the reason that consent was not obtained (e.g. the data were analyzed anonymously)<br><br>**Animal Research (involving vertebrate animals, embryos or tissues)**<br>• Provide the name of the Institutional Animal Care and Use Committee (IACUC) or other relevant ethics board that reviewed the study protocol, and indicate whether they approved this research or granted a formal waiver of ethical approval<br>• Include an approval number if one was obtained<br>• If the study involved *non-human primates*, add *additional details* about animal welfare and steps taken to ameliorate suffering<br>• If anesthesia, euthanasia, or any kind of animal sacrifice is part of the study, include briefly which substances and/or methods were applied<br><br>**Field Research**<br><br>Include the following details if this study involves the collection of plant, animal, or other materials from a natural setting:<br>• Field permit number<br>• Name of the institution or relevant body that granted permission	
**Data Availability**<br><br>Authors are required to make all data underlying the findings described fully available, without restriction, and from the time of publication. PLOS allows rare exceptions to address legal and ethical concerns. See the PLOS Data Policy and FAQ for detailed information.	Yes - all data are fully available without restriction

A Data Availability Statement describing where the data can be found is required at submission. Your answers to this question constitute the Data Availability Statement and **will be published in the article**, if accepted.<br><br>**Important:** Stating 'data available on request from the author' is not sufficient. If your data are only available upon request, select 'No' for the first question and explain your exceptional situation in the text box.<br><br>Do the authors confirm that all data underlying the findings described in their manuscript are fully available without restriction?	
**Describe where the data may be found in full sentences. If you are copying our sample text, replace any instances of XXX with the appropriate details.**<br><br>• If the data are **held or will be held in a public repository**, include URLs, accession numbers or DOIs. If this information will only be available after acceptance, indicate this by ticking the box below. For example: *All XXX files are available from the XXX database (accession number(s) XXX, XXX.).*<br>• If the data are all contained **within the manuscript and/or Supporting Information files**, enter the following: *All relevant data are within the manuscript and its Supporting Information files.*<br>• If neither of these applies but you are able to provide **details of access elsewhere**, with or without limitations, please do so. For example:<br><br>*Data cannot be shared publicly because of [XXX]. Data are available from the XXX Institutional Data Access / Ethics Committee (contact via XXX) for researchers who meet the criteria for access to confidential data.*<br><br>*The data underlying the results presented in the study are available from (include the name of the third party*	All relevant data are within the manuscript and its Supporting Information files.

and contact information or URL).<br>• This text is appropriate if the data are owned by a third party and authors do not have permission to share the data.<br><br>* typeset	
Additional data availability information:	

Powered by Editorial Manager® and ProduXion Manager® from Aries Systems Corporation

Manuscript
Click here to access/download;Manuscript;Sun_et_al_manuscript.docx

A vacuum-actuated soft robot inspired by *Drosophila* larvae to study kinetics of crawling behaviour

Xiyang Sun[1], Akinao Nose[1,2] and Hiroshi Kohsaka[1,3*]

[1] Department of Complexity Science and Engineering, Graduate School of Frontier Science, the University of Tokyo, 5-1-5 Kashiwanoha, Kashiwa, 277-8561 Chiba, Japan
[2] Department of Physics, Graduate School of Science, the University of Tokyo, 7-3-1 Hongo, Bunkyo-ku, 133-0033 Tokyo, Japan
[3] Graduate School of Informatics and Engineering, the University of Electro-Communications, 1-5-1, Chofugaoka, Chofu, 182-8585 Tokyo, Japan

* Corresponding author
E-mail: kohsaka@edu.k.u-tokyo.ac.jp

Short title: A soft robot inspired by fly larvae

Abstract

Peristalsis, a motion generated by the propagation of muscular contraction along the body axis, is one of the most common locomotion patterns in limbless animals. While the kinematics of peristalsis has been examined intensively, its kinetics remains unclear, partially due to the lack of suitable physical models to simulate the locomotion patterns and inner drive in soft-bodied animals. Inspired by a soft-bodied animal, *Drosophila* larvae, we propose a vacuum-actuated soft robot mimicking its crawling behaviour. The soft structure, made of hyperelastic silicon rubber, was designed to imitate the larval segmental hydrostatic structure. Referring to a numerical simulation by the finite element method, the dynamical change in the vacuum pressure in each segment was controlled accordingly, and the soft robots could exhibit peristaltic locomotion. The soft robots successfully reproduced two previous experimental phenomena on fly larvae: crawling speed in backward crawling is slower than forward crawling, and limiting the duration of segmental motor bursts slows down larval peristaltic motion. Furthermore, our experimental results provided novel predictions for the functional roles of kinetic parameters, including intersegmental phase delay and contraction force, in peristaltic locomotion. These observations indicate that soft robots could serve to examine the kinetics of crawling behavior in soft-bodied animals.

Introduction

Over the past few decades, robotics researchers have drawn numerous inspirations from diverse animal species to design robots (1,2). One of the recent trends to building animal-inspired robots is to utilize soft materials to construct flexible structures, mainly because the flexibility enables adaptive motions in a complex environment (2,3). Furthermore, the development of soft robots has shed light on ~~into~~ the biological mechanisms of animal motion (4,5). In particular, soft robots are useful for understanding the kinematics of soft-bodied animals' behaviours that have a high degree of freedom and complicated dynamics (6). The development of biomimetic soft robots has provided valuable platforms for both robotics and neuroscience research fields by referring to animals (7), including caterpillars (1), earthworms (8), and octopi (9).

Crawling behaviour is generated by the propagation of segmental contraction along

the body axis to move the body forward (10). Crawling is one of the basic animal motions used to move the body in one direction and has been mimicked by soft robots, including worm-like robots (8), hornworm-like robots (1), snake-like robots (11), and multigait soft robots (12). Previously, flexible braided mesh-tube structures, Meshworm and FabricWorm, have been designed based on the antagonistic muscular arrangement of earthworms (8,13). These robots use shape memory alloys and linear spring as actuators, respectively, and are capable of exhibiting crawling. However, despite these recent advances in soft robots for studying crawling behaviour, how crawling properties, including speed, are realized and regulated remains unclear (14,15).

Larvae of fruit flies, *Drosophila melanogaster*, have provided an excellent model of a soft-bodied organism to investigate peristaltic mechanisms due to their relatively simple structure, stereotyped behaviours, and accumulated knowledge of their neural circuits (16–18). The third instar fly larva is about 4 mm long and has a segmented body. The dominant larval behaviour is forward crawling, which involves the sequential translation of body segments via muscle contraction along the body axis (Fig 1; (19)). Fly larvae also exhibit backward behaviour, with the same muscles activated in the opposite sequence (20). Recently, one soft maggot robot was designed to mimic larval muscular organization and replicate larval crawling (21). It consisted of a series of pneumatic chambers that enabled body deformation by expansion. Although coordinated motions were generated, this previous maggot robot did not realize crawling behaviour.

Fig 1. Kinematic parameters in fly larval locomotion. (A) A fluorescence image of a third-instar larva expressing a green fluorescent protein in muscle attachment sites. (B) A schematic of the segment boundaries (y0-y10) and the segment names (T2-A8). (C) Displacement of the segment boundaries during larval crawling. (D) Kinematics parameters based on segmental boundary dynamics. These panels are derived from Sun *et al.* (2022).

In this work, we propose a new soft robot that can mimic larval crawling in reference to the properties of fly larvae. To mimic the contraction of body segments, a vacuum source and solenoid valves are used for actuation control. We implement an asymmetric interface between the robot and a ground substrate to enhance the forward crawling rather than

backward crawling. This larval robot successfully exhibits a crawling motion. Furthermore, the soft robot successfully reproduces two previous observations of the kinematics in fly larval crawling: slower speed in backward locomotion than in forward locomotion and significant involvement of segmental contraction duration in crawling speed. In addition, the soft robot provides predictions for the functional roles of kinetic parameters, including segmental contraction force and intersegmental phase delay. This study indicates that our vacuum-based soft robots could contribute to a better understanding of the mechanisms of crawling behaviour.

Materials and methods

By mimicking the biological properties of fly larvae, we proposed a soft robot consisting of the following three components: A) a body structure with a chain of elliptical cylinder-like segments, B) a vacuum-actuated control system, and C) software for controlling and monitoring the motion of the maggot-like soft robot. Furthermore, a simulation was conducted to model and evaluate the mechanistic properties of soft robots under different stresses.

Body structure

When designing our soft larval robot, three structural properties in fly larvae were mainly considered: 1) hydrostatic structure, 2) repetitive muscular patterning, and 3) asymmetric substrate interaction via denticle bands.

First~~ly~~, the fly larval body is filled with body fluid~~, and the~~ internal pressure and the tension of the body wall play a role in supporting their body shape. To replicate the larval hydrostatic properties, we adopted a pneumatic structure with silicone rubber among the current soft actuator candidates (2,22). Secondly, the configuration of muscles in the body wall is segmentally repeated in fly larvae. The larval body consists of 11 segments: three thoracic and eight abdominal segments. Although the terminal segments (the first thoracic (T1) and the last abdominal (A8) segments) have specialized structures, the other nine segments have an almost consistent structure. The larval length and width were examined as 3.69 ± 0.56 mm and 0.66 ± 0.09 mm (23). The length-to-width ratio of a single segment in

the major middle segments is about 0.5 (Fig 1). To mimic the larval shape, the individual segment was designed as an elliptical cylinder chamber with a flat plane at the bottom. We constructed two soft robots with different segmental length-width ratios: The width of each segment was 30 mm, whereas the axial length of the segments of the three robots was 20 mm and 30 mm, respectively. Accordingly, their length-to-width ratios of them were 2/3 and 1, respectively. To simplify the robot while allowing us to analyze the propagation of segmental contraction, we set the number of the segments in one robot as five (Fig 2). Finally, spike-like structures, named denticle bands, align at the bottom of each segment in the fly larvae. Denticle bands act as anchorage points to the ground during the propagation of segmental deformation (24). Each denticle band normally consists of six rows of denticles in a larva. The hooked tips of four out of the six rows point posteriorly while the remaining two rows point anteriorly (24), suggesting that the friction between the ventral body surface and the ground substrate differs between forward and backward motions. To mimic this asymmetric denticle structure between anterior and posterior directions, we implemented an asymmetric friction structure by gluing a piece of paper (Whatman paper 1001-917) at the anterior side of the segment boundary (shown as the thick lines in Supplementary Fig 1C). Silicone rubber was stickier than paper; hence the physical contact between the silicone rubber and a ground substrate would generate larger friction than the one between paper and the ground substrate. By virtue of this property and the geometric fact that the angle of the segment boundary depended on the direction of the propagation of segment contraction, asymmetric friction during locomotion in different directions could be realized.

Fig 2. The fabrication process for the soft robot. (A) Moulds printed by a 3D printer. (B and C) Preparation for a segmental chamber of the soft robot. (D and E) Assembling segmental chambers to build the soft robot. (F) The soft robot was connected to a pneumatic pump via air tubes.

We fabricated soft robots based on the design shown in Fig 2. Firstly, the moulds for the soft robots were printed with Acrylonitrile Butadiene Styrene (ABS) material with a 3D printer (WANHAO Duplicator 4S). Then, the moulds were assembled (Fig 2A), and silicone rubber (Ecoflex 00-30) was poured to fill the interior space between the inner and the outer

moulds. After the gap was filled with silicone rubber, a cross-section-sized piece of paper (Whatman paper 1001-917) was placed on the top surface of the silicone rubber (Fig 2B). The rubber was cured in a drying oven (at 80℃ for about 20 minutes) and cooled down at room temperature. Afterwards, the single segment structure was demoulded, as shown in Fig 2C. Finally, five segmental structures were glued together using the Ecoflex mixture to build the final structure (Fig 2D and 2E). A fabricated soft robot is shown in Fig 2F.

Vacuum control system

To enable the segmental chambers to contract like body segments in fly larvae, we took advantage of a vacuum-based actuator. We built a system consisting of pneumatic circuits, electrical circuits (dashed and solid lines respectively in Fig 3), and a graphical user interface software (implemented on "Computer" in Fig 3).

Fig 3. The framework of the whole system. The control system for the soft robots is composed of electrical connections (solid lines) and pneumatic connections (dashed lines).

The pneumatic circuits were established to control the pressure in each segment chamber. Unlike previous larvae-like robots based on the segmental expansion (21), we adopted contraction as a driving force to mimic the muscular contraction of fly larvae in this study. Pneumatic pathways linked a vacuum source (TAITEC VC-15s, with a pressure range from -110kPa to 0kPa), three-way solenoid valves (ZHV 0519), vacuum pressure sensors (MPXV6115V), and robotic chambers (see “Body structure” in the Materials and methods section). Each chamber and the vacuum source were connected through a vinyl tube and a solenoid valve. The tube was flexible and lightweight (1.4 g) compared with the soft robots (16.9 – 29.9 g). Even when we held the tubes, the motion of the soft robots was not disturbed, showing that the tube had little effect on the kinematics of the soft robot. The pressure within each chamber was regulated by gating these solenoid valves.

Electrical circuits were built to control the pressure valves for individual chambers. A microcontroller (Arduino Mega) was selected considering the number of PWM pins and the output voltage. The microcontroller was connected to solenoid valves and vacuum pressure sensors to regulate and monitor robot deformation. The microcontroller's detailed electrical

circuits are presented in Supplementary Fig 2, including modules for the solenoid valves and vacuum sensors. Here, an NPN transistor (TIP120) modulates an external high-power source to drive the solenoid valves based on the PWM signal from the microcontroller. The 6V external power, provided via a DC-DC power supply regulator (ARD-PWR), was the maximal operational voltage for the solenoid valves. Since the valves possessed non-negligible inductance, diodes (1N4007) were used in a flyback configuration to prevent a large back-electromotive force (back emf), which could damage these valves. These diodes could dissipate the remaining energy. The vacuum pressure, in the range of –115 to 0 kPa relative to the standard atmosphere, was measured by the output of the voltage-based vacuum sensor based on the following equation:

$$P = \frac{V_{OUT} - 0.92 \cdot V_S}{0.007652 \cdot V_S} \pm c_{Temp} \cdot P_{error}$$

where P is the vacuum pressure, and V_{OUT} and V_S represent the output voltage and voltage supply, respectively, in the pressure sensor. P_{error} is the pressure error, which indicates the measurement error in the pressure at a standard temperature (P_{error} is in units of pressure.) Dimensional parameter c_{Temp} is a temperature factor for the pressure error, which is 1 within the temperature ranges from 0 to 85℃. According to the sensor datasheet, the maximum value of P_{error} was 1.725 kPa within the range of our experiments. Low-pass filters were applied to pressure signals from vacuum sensors (Supplementary Fig 2).

Software for controlling and monitoring soft robot locomotion

To monitor the position of the segment boundaries and their segmental pressures in operation in real-time, we designed a graphical user interface (GUI) (Supplementary Fig 3) based on Python libraries (Tkinter and pySerial). The USB camera (HOZAN camera with 12mm lens) was attached to a bracket right above the soft robot. Its frame rate was 40 frames per second, used in the following experiments. The images were sent to the computer via a USB connector, and the robotic motion could be observed on the GUI (Fig 4 right, Supplementary Fig 3 left). Meanwhile, the vacuum pressures in the segmental chambers were monitored in the Sensor Signal panel of the GUI window (Supplementary Fig 3 right). The position of the front end ("head") and rear end ("tail") was measured by a ruler shown in Fig 4. Example locomotion and pressure signals are shown in Supplementary Fig 3.

Fig 4. System overview. The soft robot system includes the following: the soft structure, electrical circuits, the vacuum source and pressure gauge, a motion platform, a USB camera and a PC with a GUI window.

Simulation

To determine the suitable ranges of the pressure and time scale for robot deformation, we applied the finite element method (FEM) to simulate our soft robot via the commercial FEM software (Abaqus). To achieve a reliable simulation, suitable viscoelastic physical models were required. In our project, the main soft body was constructed using hyperelastic material (Ecoflex 00-30). In line with previous modelling and validation work (6), the Ogden model was adopted to model this material. The strain energy potential function U, which describes the elastic properties of the material, is defined as:

$$U(\lambda_1, \lambda_2, \lambda_3, J) = \sum_{i=1}^{N} \frac{2\mu_i}{\alpha_i^2}\left(\lambda_1^{\alpha_i} + \lambda_2^{\alpha_i} + \lambda_3^{\alpha_i} - 3\right) + \sum_{j=1}^{N} \frac{1}{D_j}(J-1)^{2j} \quad (2)$$

where μ_i and α_i $(i = 1, 2, 3)$ are the primary fitting parameters, and λ_j $(j = 1, 2, 3)$ are the stretches along the x, y, and z axes. The material constant N was set as three. The second summation term contains fitting parameters D_j $(j = 1, 2, 3)$ to the volumetric deformation and the material Jacobian matrix J. We referred to the previous parameters for this hyperelastic material (6). Meanwhile, regarding the paper (Whatman paper, see “Body structure” in the Materials and methods section) on the intersection of soft robots, the material density was 0.483 mg/mm^3, and its young's module was 1.71GPa, and the Poisson ratio was -0.3 (25). Considering the segmental deformation under vacuum pressure, we configured the self-contact condition for the interaction step.

We calculated the responses of the soft robot under distinct pressures using the FEM software Abaqus (Fig 5). With respect to the nonlinear dynamics within our model, we configured parameters for incrementation to ensure a successful simulation: the maximum number of increments as 100 and the minimum increment size as 1e-15. Here, the example soft robot with five 20 mm segments was indicated as 20mmx5-SR (20 mm, five segments, and Soft Robot), and a similar shorthand notation was used for 30 mm segments 30mmx5-SR. Two points at the bottom of the terminal segment (labelled “Head” and “Tail” in Fig 5A) were used to monitor the head and tail segmental position in the longitudinal axis to record their asymmetric deformation. When the negative pressure of -10kPa was applied to the most

anterior chamber (head chamber) of 20mmx5-SR, the head marker exhibited a negative displacement (Fig 5B), which indicated that the head segment was contracted, and the end of the head moved backwards. The head marker moved faster when a larger absolute value of the negative pressure (from -20kPa to -70kPa) was applied to the head chamber. This observation indicated that the kinematics of the segment dynamics could be regulated by the pressure within the soft robot chambers. Since there was no asymmetricity along the body axis in the FEM simulation, when negative pressures were applied at the most posterior chamber (tail chamber), the tail marker showed similar displacements but in the opposite direction (Fig 5C). The larger soft robot (30mmx5-SR) exhibited faster segment contraction (Fig 5D and 5E). In either case, the movement was carried out in less than 1 sec, comparable to the stride duration in fly larval locomotion.

Fig 5. Simulation of the soft robots by FEM. (A) Section view of the soft robot generated in Abaqus. The displacements of the head and tail were monitored. (B-C) Displacements of the head (B) and tail (C) of the 20mmx5 soft robot during the simulated contraction of the corresponding segment. (D-E) Displacements of the head (D) and tail (E) of the 30mmx5 soft robot during the simulated contraction of the corresponding segment. The internal pressures were tested from -70 kPa to -10 kPa.

Statistics

Statistical calculation was conducted by Python 3.7. P-values in one-way ANOVA tests and Welch's t-test were calculated by the SciPy library.

Results

Control of the chambers in the soft robots by vacuum

In this study, we analyzed the locomotion of the soft robots under conditions with varied contraction forces and distinct intersegmental phases. Since the pressure of the vacuum source was constant, we tried to realize varied pressures by controlling the duty cycle of the gating of solenoid valves. To this aim, we adopted the Pulse-Width-Modulation (PWM) method (11) to control the temporal patterns of the chamber pressures. In this method,

a series of pulses are generated, and the frequency and the duty cycle of the pulses can be tuned. We set the frequency as 1000 Hz because it was fast enough to reproduce fly larval crawling that occurred on the scale of 100 ms. The timer frequency of 1000 Hz was realized by setting a parameter OCRnA for the Arduino Mega board as 249 (= 16 MHz / 1000 Hz / 64 – 1). On the other hand, the duty cycle could be adjusted by OCRnB, another parameter in the timer. The default range of OCRnB is from 0 to 255 (Fig 6A). OCRnB set the value to threshold a sawtooth timer signal to produce 1 kHz PWM signals with varied duty cycles (Fig 6A). We then used the resulting PWM signal to control segmental deformation via vacuum pressure. The deformation of the head segment was monitored under different duty cycles (Fig 6B). The results showed that the segmental deformation could not be observed when the OCRnB is smaller than 190, which means that the solenoid valve doesn't work in this case. On the other hand, when OCRnB was 195 or more, the contraction of the head segment was observed. In particular, as OCRnB increased, the speed of the deformation increased, which suggested that the contraction force within the head segment chamber was higher when the duty cycle was larger. Accordingly, we succeeded in temporally controlling the pressures within the segments of the soft robot. Even when we changed the waveform from the square to others, such as sinusoidal and saw-like waveforms, the temporal profiles of chamber pressure were similar to that with a square waveform input (Supplementary Fig 4). Then we decided to use the square waveform to control the chamber pressure in the following analyses.

Fig 6. Testing suitable parameter configurations in the PWM control. (A) Schematic for the fast PWM mode. The level of OCRnA and OCRnB was set to control the frequency and duty cycle of the PWM signal, respectively. OCRnA was set to 249 to ensure a time-frequency of 1000 Hz. (B) Deformation of single segments with distinct duty cycles set by OCRnB.

Referring to the results from the FEM simulation (see Simulation in the Materials and methods section), we analyzed the response of the soft robots to the pneumatic operation (Fig 7). When negative pressure was applied to the head chamber, the head marker on the 20mmx5-SR exhibited negative displacements, which was consistent with the FEM simulation results (Fig 7B). The displacement gradually reached a stable value in less

than 1 second. A similar tendency was observed when negative pressures were applied to the tail chamber (Fig 7C). The larger soft robot (30mmx5-SR) showed weaker displacement than 20mmx5-SR (Fig 7D and 7E). All these observations in the measurement in the soft robots were consistent with the FEM results. These analyses indicated the larger mass of soft robots, such as 30mmx5-SR, would make the robot harder to deform and move. Hence, we described experimental results for the 20mmx5-SR soft robot in the following sections.

Fig 7. Deformation of the terminal segments of the soft robots by pneumatic control. (A) The displacements of the head and tail of the soft robots were monitored. (B-C) Displacements of the head (B) and tail (C) of the 20mmx5 soft robot by pneumatic control of the corresponding segment (head or tail segment). (D-E) Displacements of the head (D) and tail (E) of the 30mmx5 soft robot by pneumatic control of the corresponding segment (head or tail segment). The internal pressures were tested from -70 kPa to -10 kPa.

One striking difference between the simulation and the soft robot experiment was the existence of asymmetric friction. As described in Supplementary Fig 1C, the friction at the interface between the soft robot and the ground substrate was asymmetric. Due to this effect, the tail marker showed a slightly larger displacement upon applying negative pressure to the tail chamber than the head marker did upon the negative pressure applied to the head chamber. (Fig 7).

In summary, we implemented and optimized three properties in the soft robot to mimic fly larvae: The contraction of chambers instead of the expansion, which was used previously (21), the asymmetric feature of the interface between the soft robot and the ground substrate, and optimal ranges in pressure, time scale, and the size of the chambers.

Robotic locomotion and its quantification

Next, the crawling ability of the soft robot was tested. Using the PWM method, we generated temporal patterns coding that negative pressure was sequentially applied from the posterior to anterior chambers (Fig 8). As an initial case, we set the vacuum pressure as – 10 kPa, segmental contraction duration as one second, and overlap of the contraction time between neighboring segments as zero (Fig 8A). Under this condition, the soft robot exhibited

a crawling motion and could move forward (Fig 8B). Accordingly, this result suggests that our soft robot could provide a physical model to analyze the crawling behavior. In the following sections, we attempted to reproduce two previous experimental results and make one new prediction on fly larval locomotion with the soft robot.

Fig 8. Performance of the soft robots. (A) Schematic of the control signal for the soft robot to drive forward crawling. (B) Still images of soft robot locomotion. The soft robot crawls from left to right.

Asymmetric speed between forward and backward crawling

As the first experimental observation in fly larvae, we focused on the difference between forward and backward crawling speed. A previous study showed fly larvae move faster by forward crawling than backward (26). However, the possible contribution of the difference in the body-ground friction during forward and backward motion to the different speeds between forward and backward crawling has not been tested. By using our soft robot, we tested this possibility. The displacements of the soft robot were calculated based on the position of its front end (“Head” in Fig 7A). The speed of robot crawling was measured using five successive strides. To generate backward crawling, we controlled the spatiotemporal pattern of the chamber pressures in a similar way to that of forward crawling (Fig 9A) but in reverse order (Fig 9B). We scanned the maximum pressures, stride durations, and intersegmental delays and compared the speed of crawling between backward and forward locomotion. In all the cases, backward crawling was slower than forward crawling (Fig 9C). This observation implies that the asymmetric properties of the interface between the larval body and the ground substrate could be a critical factor in the asymmetric crawling speed between forward and backward movements.

Fig 9. Forward and backward crawling in the soft robot. (A and B) Control signals for forward (A) and backward (B) locomotion of the soft robot. (C) Locomotion speed of the soft robot in forward and backward locomotion. Forward crawling was faster than backward crawling (p-value = 9.3×10^{-5} by Welch's t-test).

Involvement of segmental contraction duration and intersegmental phase delay in locomotion speed

We next analyzed the relationship between the segmental kinematics and crawling speed. A previous study reported that a class of inhibitory interneurons (PMSIs, period-positive median segmental interneurons) was involved in crawling speed in fly larvae (27). When the activity of PMSIs was blocked, the segmental contraction duration and the delay in contraction between neighboring segments were elongated. Furthermore, the larvae with reduced PMSI activity exhibited slower crawling. These observations suggested that the segmental contraction duration or the intersegmental phase delay should be involved in crawling speed, but this hypothesis has not been tested. Taking advantage of our soft robot, we investigated this possibility.

First, we changed the segmental contraction duration while keeping the minimum pressure constant (-30 kPa) and fixing the intersegmental phase delay (60%) (Fig 10A and 10B; We defined intersegmental phase delay as the ratio of an intersegmental time delay to segmental contraction duration. See below for details) The result showed that the soft robot with a shorter segmental contraction duration (up to 0.2 sec) exhibited faster crawling (Fig 10C). This observation was consistent with the observation in the loss of function experiment of PMSIs.

Fig 10. Involvement of segmental contraction duration and intersegmental phase delay in crawling speed. (A and B) Control signals with different segmental contraction duration (double-headed arrows) for the soft robot. (A) The segmental contraction duration is 0.4 sec. (B) The segmental contraction duration is 0.8 sec. The intersegmental phase delay is the same in (A) and (B). (C) Locomotion speed of the soft robot with different segmental contraction duration (p-value = 1.1×10^{-4} by one-way ANOVA). (D and E) Control signals with different intersegmental phase delays (double-headed arrows) for the soft robot. (D) The intersegmental phase delay is 60%. (E) The intersegmental phase delay is 100%. The segmental contraction duration is the same in (D) and (E). (F) Locomotion speed of the soft robot with a different intersegmental phase delay (p-value = 0.015 by one-way ANOVA).

Next, we perturbed the intersegmental phase delay while keeping other conditions

(Fig 10D and 10E). In fly larvae, the contraction of neighboring segments overlaps during larval crawling (17). The stride duration we measured from fly larvae was about one second, and the average segmental contraction duration was around 0.5 sec. The intersegmental delay was obtained by dividing the stride duration (1 sec) by the number of segments (10), giving a 0.1-second intersegmental delay. Accordingly, the phase delay of neighboring segments in fly larvae was 20% (= 0.1 sec / 0.5 sec). We investigated the effects of segmental phase delay on crawling speed, which has not been examined before.

We measured robot locomotion with various segmental phase delays. For our five-segment robot, 20% and 40% phase delays were too short to generate stable locomotion because all the segments were shrunk under these conditions. To make time for segments to be relaxed, we tested segmental phase delays of 60%, 80%, and 100% while keeping segmental contraction time constant (0.4 sec) and fixing the minimum pressure (-30 kPa). We found that as the segmental phase delay increased, the crawling speed became slower (Fig 10F). These phenomena would be because the smaller segmental phase delay promoted cooperative contraction of neighboring segments, leading to larger contraction of segments and faster crawling speed. To sum, the perturbation experiments with our soft robot showed that both the segmental contraction duration and the intersegmental phase delay could be involved in crawling speed, consistent with the previous experimental observation.

Prediction of the relationship between the maximum contraction force and locomotion speed

Finally, we analyzed the relationship between segmental contraction force and the crawling speed, which has not yet been examined in fly larvae. We systematically changed the vacuum pressure from –10 kPa to –70 kPa while keeping the segmental contraction duration constant (one second corresponding to the segmental contraction time of 0.2 sec for a five-segment soft robot) and no overlapping between neighboring segmental contractions. The results showed that the soft robot operated with larger absolute values of the negative pressure and exhibited a faster crawling speed (Fig 11). This tendency could be observed in different segmental contraction durations. This observation could provide a prediction that a larger muscular force could generate faster crawling in fly larvae.

Fig 11. Relationship between the contraction force and crawling speed. (A) Control signals for forward locomotion of the soft robot. (B) Locomotion speed of the soft robot with different vacuum pressure amplitudes (p-value = 2.6 x 10^{-7} by one-way ANOVA).

Discussion

In this work, we developed a new vacuum-actuated soft robot to mimic fly larval locomotion. Our soft robot could show peristaltic locomotion patterns produced by the propagation of segmental contraction waves. Two points were crucial to realizing effective peristaltic locomotion in our soft robots: First, pieces of paper inserted in the cross-section help generate asymmetric friction between the soft robot and ground substrate, resulting in different locomotion speeds in different directions. Currently, designs for asymmetric friction in soft robots have still been paid little attention. The simple but effective implementation using paper in our robot implied the importance of friction asymmetricity. Second, to determine the control signals for the soft robot, we used the kinematic properties in fly larval crawling and configured various kinetic parameters, including pressures, stride durations, and intersegmental phase delays. Based on the robotic locomotion, we found that the contraction force, intersegmental phase delay, and stride duration all contributed to the regulation of crawling speed.

Analyses using our soft robots were consistent and gave novel interpretations to previous works. The mechanism of PMSI neurons indicated that shorter intersegmental phase delays promoted faster speed. Assays using soft robots provided evidence (Fig 10) consistent with this. Furthermore, the observation that our soft robot crawled faster in the forward than backward direction was consistent with the previous kinematic study (26). According to the present study, the difference in speed between forward and backward crawling would be partially attributed to the asymmetric friction property between forward and backward directions. Since two separate neural circuits are involved in forward and backward crawling in the central nervous system (20), the different forward and backward crawling speeds might be realized by two parallel mechanisms: friction asymmetricity and direction-specific neural circuits.

There are several ways to improve the performance of robotic crawling in future

studies. First, the physical properties of soft materials are critical for the dynamics of soft robots. The crawling speed should be improved by utilizing soft materials with larger frictional coefficients for the soft robot. Second, a better actuation source, which can make the soft robot untethered, would broaden its applications in practical scenarios. Third, implementing soft sensors on the robotic body can make it more bionic. It has been reported that the proprioception of the body wall is key to generating an innate crawling speed in *Drosophila* larvae (28). By monitoring the deformation on the surface of the soft robot and using this information to control the crawling behavior, the soft robot could exhibit flexible and adaptive locomotion in various complicated environments. Last but not least, although we tested two different sizes of robots, the scale of the robot can still be modified. By changing the number and size of the segments, locomotion ability could be changed. By optimizing these conditions, the application range of our soft larval robots would be broadened.

The kinematic results in our soft robot system have the potential to inspire further study of larval motor outputs, including the mechanisms and kinematic effects of segmental contraction force and phase delay. And our attempts to better understand the mechanisms in soft-bodied animals will contribute to designing adaptive and robust soft robots.

Acknowledgements

This work was supported by MEXT/JSPS KAKENHI grants (17K19439, 19H04742, and 20H05048 to AN and 17K07042 and 20K06908 to HK). We thank Drs Jane Loveless and Dai Owaki for their critical comments on our manuscript.

References

1. Umedachi T, Kano T, Ishiguro A, Trimmer BA. Gait control in a soft robot by sensing interactions with the environment using self-deformation. R Soc Open Sci. 2016 1;3(12).
2. Boyraz P, Runge G, Raatz A. An overview of novel actuators for soft robotics. High Throughput. 2018;7(3):1–21.
3. Aguilar J, Zhang T, Qian F, Kingsbury M, McInroe B, Mazouchova N, et al. A review on locomotion robophysics: The study of movement at the intersection of robotics, soft

matter and dynamical systems. Reports on Progress in Physics. 2016;79(11).
4. Zhang J, Wang T, Wang J, Li B, Hong J, Zhang JXJ, et al. Dynamic modeling and simulation of inchworm movement towards bio-inspired soft robot design. Bioinspir Biomim. 2019;14(6):066012.
5. Wehner M, Truby RL, Fitzgerald DJ, Mosadegh B, Whitesides GM, Lewis JA, et al. An integrated design and fabrication strategy for entirely soft, autonomous robots. Nature. 2016;536(7617):451–5.
6. Moseley P, Florez JM, Sonar HA, Agarwal G, Curtin W, Paik J. Modeling, Design, and Development of Soft Pneumatic Actuators with Finite Element Method. Adv Eng Mater. 2016;18(6):978–88.
7. Webb B. From insects to robots. Arthropod Struct Dev. 2017;46(5):687–8.
8. Kandhari A, Mehringer A, Chiel HJ, Quinn RD, Daltorio KA. Design and actuation of a fabric-based worm-like robot. Biomimetics. 2019;4(1).
9. Giorelli M, Renda F, Calisti M, Arienti A, Ferri G, Laschi C. A two dimensional inverse kinetics model of a cable driven manipulator inspired by the octopus arm. Proceedings - IEEE International Conference on Robotics and Automation. 2012. p. 3819–24.
10. Seok S, Onal CD, Wood R, Rus D, Kim S. Peristaltic locomotion with antagonistic actuators in soft robotics. Proc IEEE Int Conf Robot Autom. 2010;0060:1228–33.
11. Crespi A, Badertscher A, Guignard A, Ijspeert AJ. AmphiBot I: An amphibious snake-like robot. Rob Auton Syst. 2005;50(4):163–75.
12. Shepherd RF, Ilievski F, Choi W, Morin SA, Stokes AA, Mazzeo AD, et al. Multigait soft robot. Vol. 108, Proceedings of the National Academy of Sciences. 2011. p. 20400–3.
13. Seok S, Onal CD, Cho KJ, Wood RJ, Rus D, Kim S. Meshworm: A peristaltic soft robot with antagonistic nickel titanium coil actuators. IEEE/ASME Transactions on Mechatronics. 2013;18(5):1485–97.
14. Robertson MA, Paik J. New soft robots really suck: Vacuum-powered systems empower diverse capabilities. Sci Robot. 2017;2(9):1–12.
15. Ijspeert AJ. Annual Review of Control, Robotics, and Autonomous Systems Amphibious and Sprawling Locomotion: From Biology to Robotics and Back. Annu Rev Control Robot Auton Syst. 2020;
16. Clark MQ, Zarin AA, Carreira-Rosario A, Doe CQ. Neural circuits driving larval locomotion in Drosophila. Neural Dev. 2018;13(1):1–10.
17. Kohsaka H, Guertin PA, Nose A. Neural Circuits Underlying Fly Larval Locomotion. Curr Pharm Des. 2017;23(12):1722–33.
18. Gowda SBM, Salim S, Mohammad F. Anatomy and neural pathways modulating

distinct locomotor behaviors in Drosophila larva. Biology (Basel). 2021;10(2):1–30.

19. Sun X, Liu Y, Liu C, Mayumi K, Ito K, Nose A, et al. A neuromechanical model for Drosophila larval crawling based on physical measurements. BMC Biol. 2022;20(1):130.

20. Kohsaka H, Zwart MF, Fushiki A, Fetter RD, Truman JW, Cardona A, et al. Regulation of forward and backward locomotion through intersegmental feedback circuits in Drosophila larvae. Nat Commun. 2019;10(1).

21. Wei T, Stokes A, Webb B. A soft pneumatic maggot robot. In: Conference on Biomimetic and Biohybrid Systems. Springer Verlag; 2016. p. 375–86.

22. Bhagat S, Banerjee H, Tse ZTH, Ren H. Deep reinforcement learning for soft, flexible robots: Brief review with impending challenges. Robotics. 2019;8(1):1–27.

23. Ormerod KG, LePine OK, Abbineni PS, Bridgeman JM, Coorssen JR, Mercier AJ, et al. Drosophila development, physiology, behavior, and lifespan are influenced by altered dietary composition. Fly (Austin). 2017;11(3):153.

24. Bejsovec A. Wingless/Wnt signaling in Drosophila: The pattern and the pathway. Vol. 80, Molecular Reproduction and Development. John Wiley & Sons, Ltd; 2013. p. 882–94.

25. Fernandes SC, Walz JA, Wilson DJ, Brooks JC, Mace CR. Beyond Wicking: Expanding the Role of Patterned Paper as the Foundation for an Analytical Platform. Anal Chem. 2017;89(11):5654–64.

26. Heckscher ES, Lockery SR, Doe CQ. Characterization of Drosophila Larval Crawling at the Level of Organism, Segment, and Somatic Body Wall Musculature. Vol. 32, Journal of Neuroscience. 2012. p. 12460–71.

27. Kohsaka H, Takasu E, Morimoto T, Nose A. A group of segmental premotor interneurons regulates the speed of axial locomotion in drosophila larvae. Current Biology. 2014;24(22):2632–42.

28. Hughes CL, Thomas JB. A sensory feedback circuit coordinates muscle activity in Drosophila. Molecular and Cellular Neuroscience. 2007;35(2):383–96.

Author contributions

XS and HK conceived this project. XS conducted the simulation, built the soft robots, collected and analyzed the data, and wrote the manuscript. AN edited the manuscript. HK supervised the work, analyzed the data, and wrote the manuscript. All authors read and approved the final manuscript.

Data availability

All data is included in the manuscript and Supporting information.

Competing interests

The authors declare no competing interest.

Supporting information

Supplementary Fig 1. Design of the soft robot. (A) Mould sketch for the soft structure. (B) The segmentally-repeated structure of the soft robot. (C) Locomotion scheme with segmental deformation. Red circles show the asymmetric friction between anterior and posterior segmental boundaries in body-substrate interaction. The left is anterior. Sheets of paper inserted in the soft robot are represented by thick lines.

Supplementary Fig 2. Illustration of the electrical circuits. The left part represents the main connections for solenoid valves and pressure sensors. The right parts show the solenoid valve module (top) and pressure sensor module (bottom).

Supplementary Fig 3. The GUI window for recording the locomotion of the soft robot.

Supplementary Fig 4. Single-segment deformations in 20mmx5SR by different control signals. (A - E) The upper panels represent different control signals: square (A), sinusoid (B), and saw (C - E) waveforms. The middle panels show the pressure measured in the segmental chambers. The bottom panels represent the segment deformation induced by the control signal shown in the upper panels.

Click here to access/download;Figure;Fig1.tif

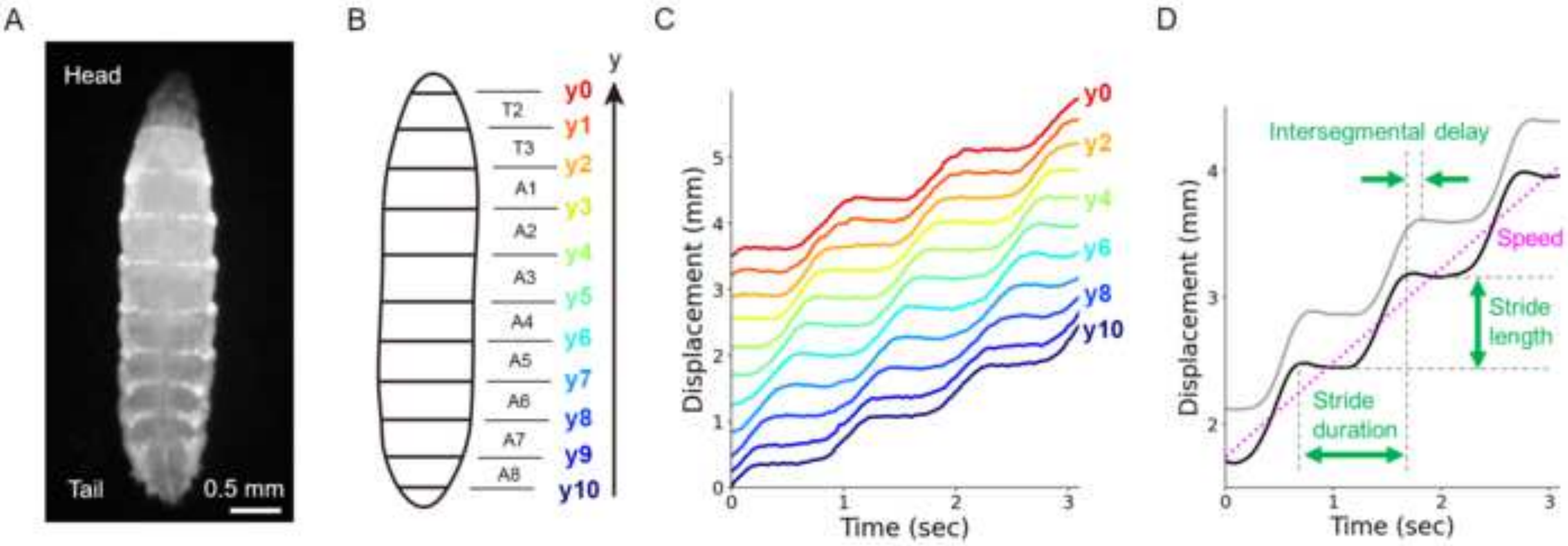

Click here to access/download;Figure;Fig2.tif

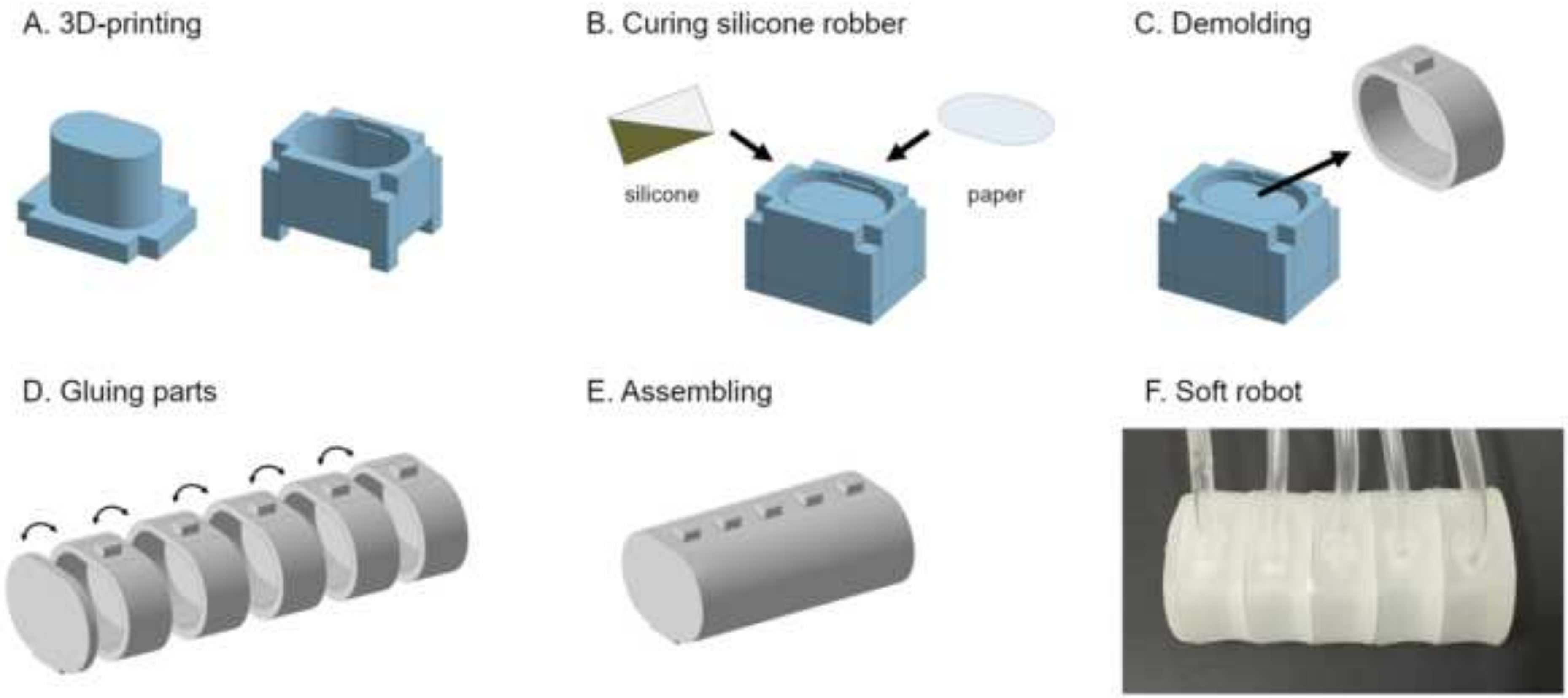

Fig3

Click here to access/download;Figure;Fig3.tif

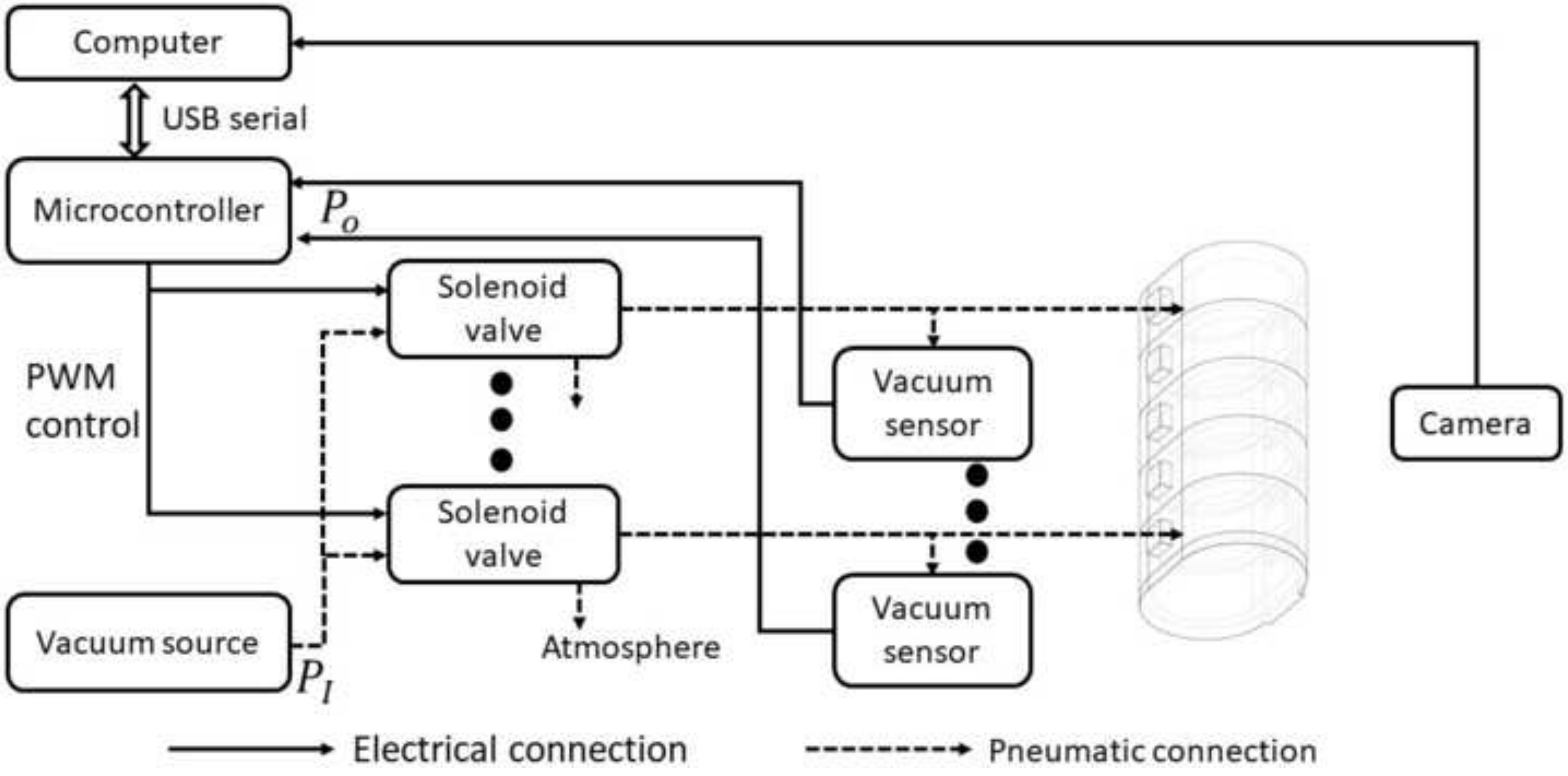

Fig4

Click here to access/download;Figure;Fig4.tif

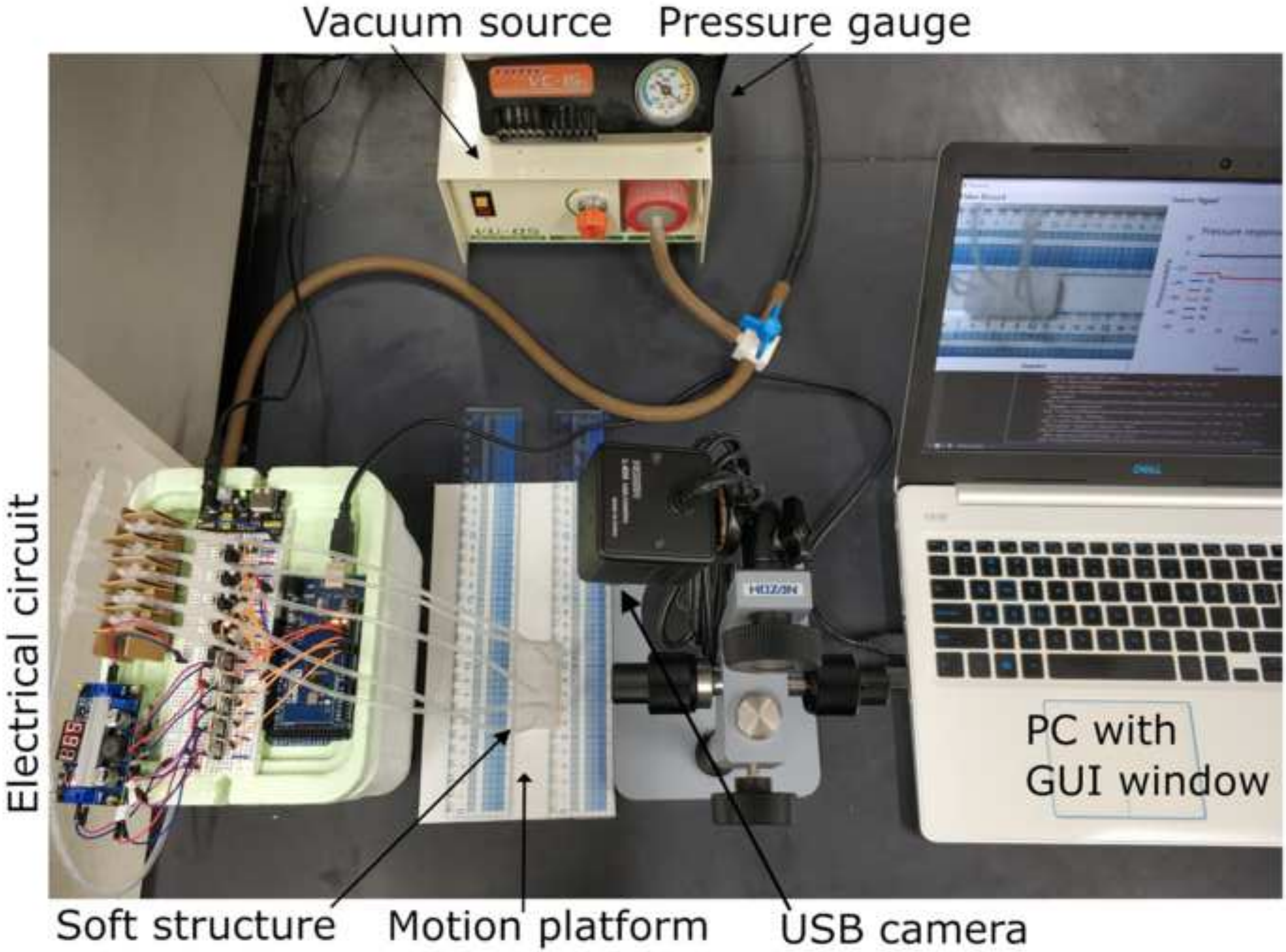

Fig5

Click here to access/download;Figure;Fig5.tif

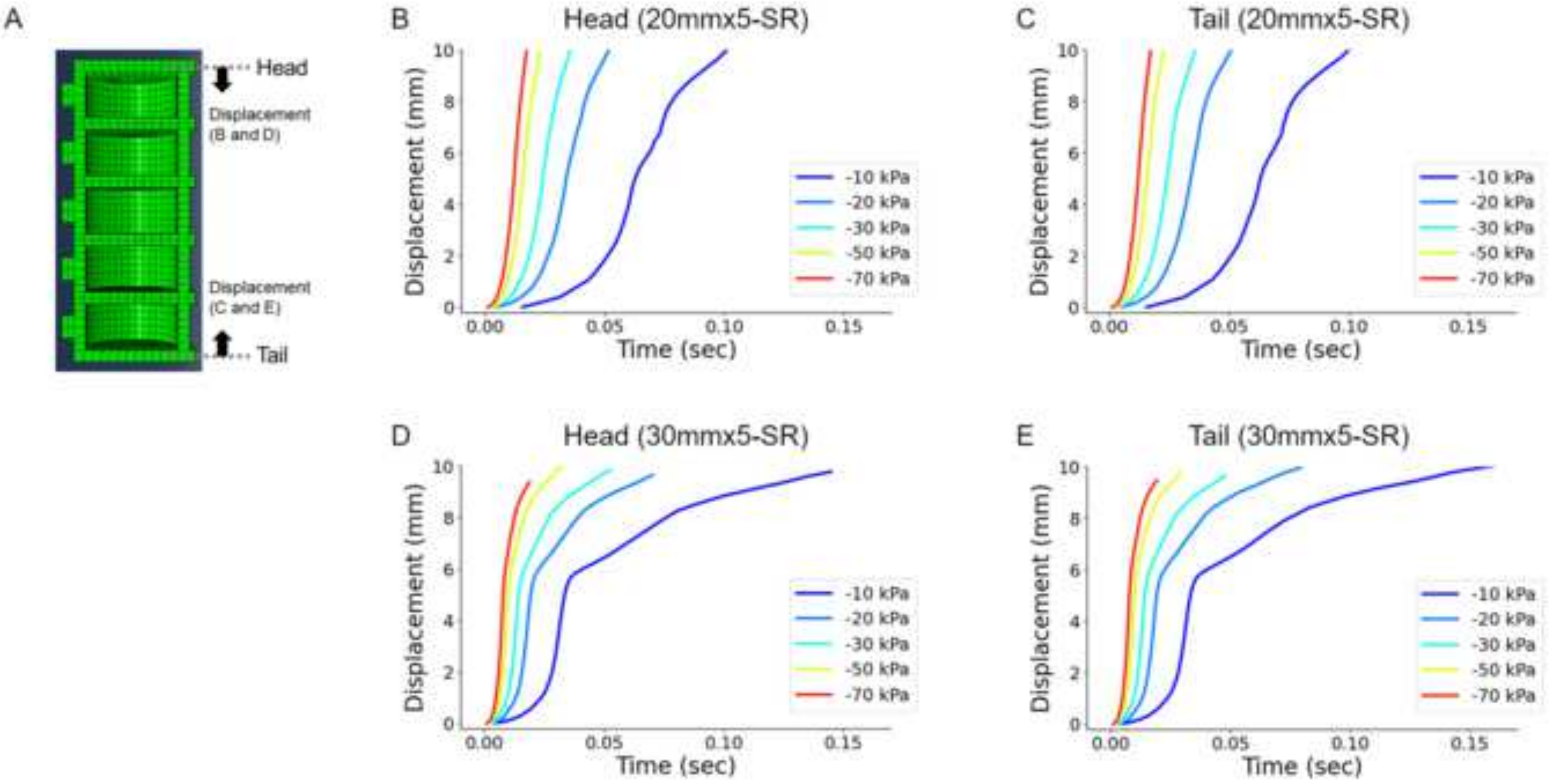

Fig6

Click here to access/download;Figure;Fig6.tif

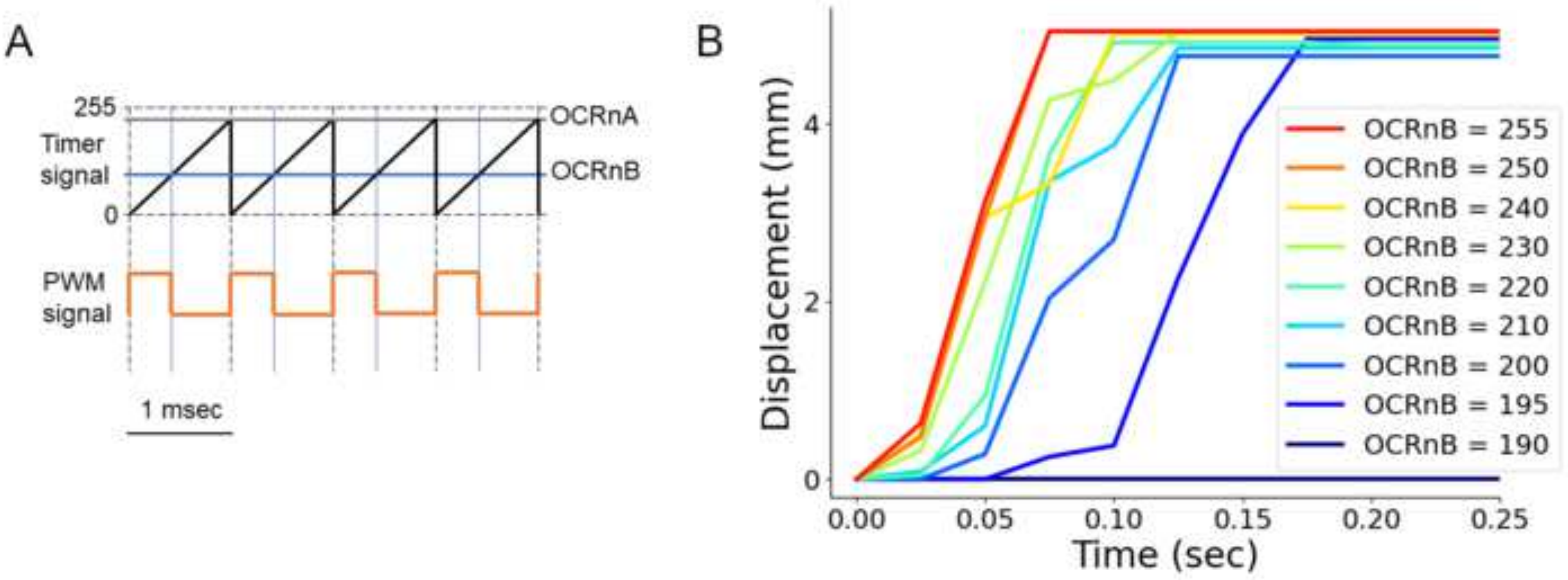

Fig7

Click here to access/download;Figure;Fig7.tif

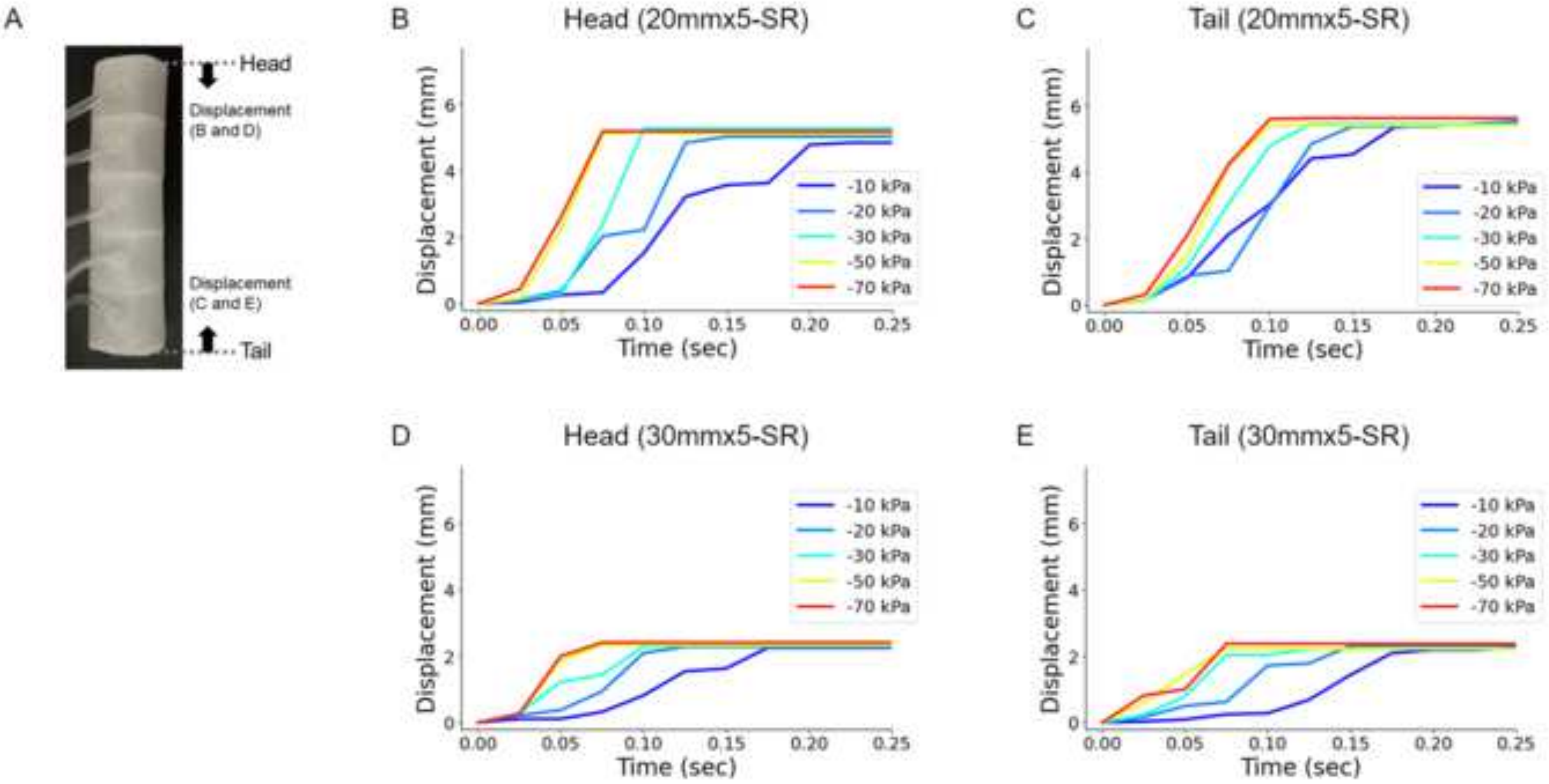

Fig8

Click here to access/download;Figure;Fig8.tif

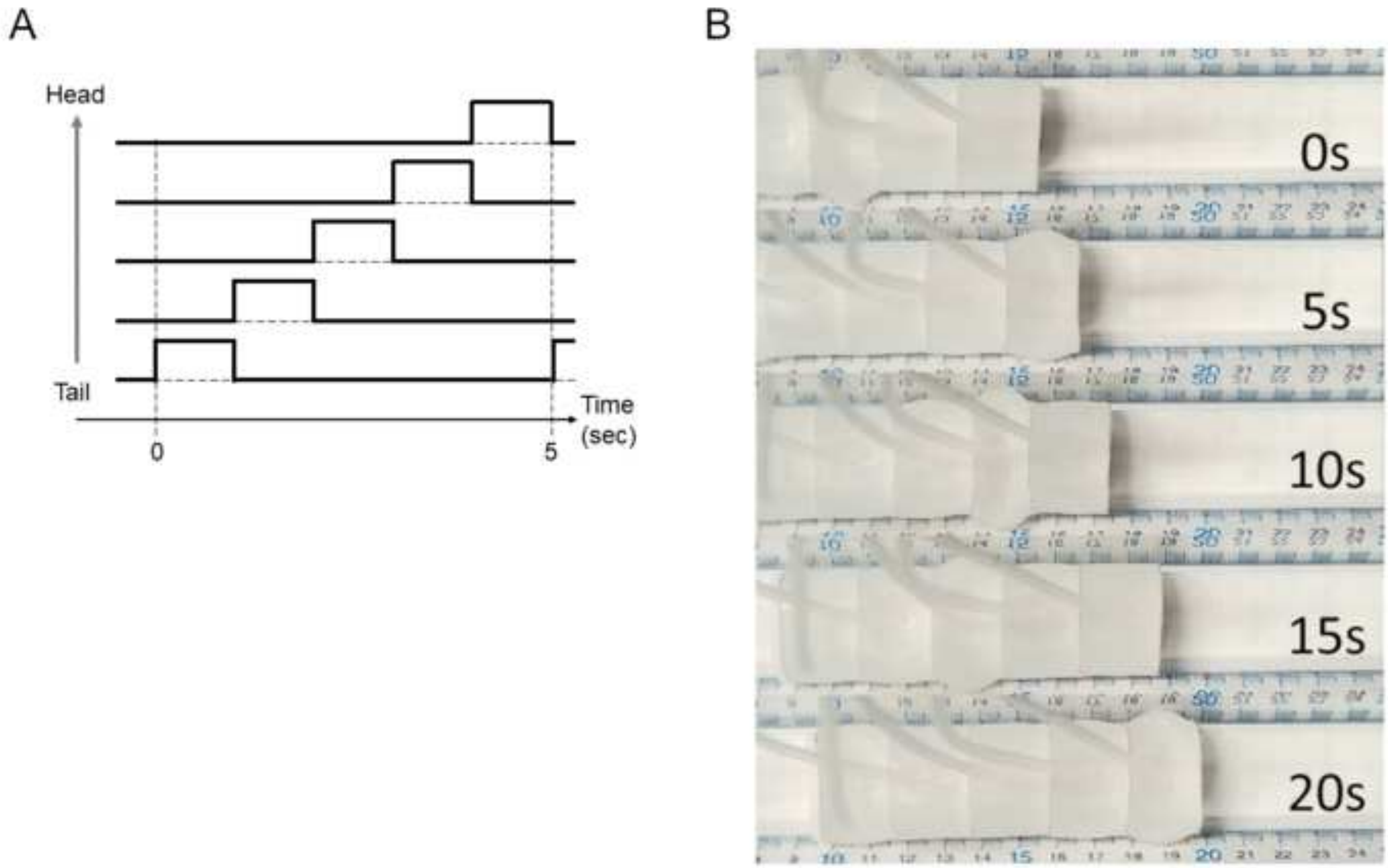

Click here to access/download;Figure;Fig9.tif

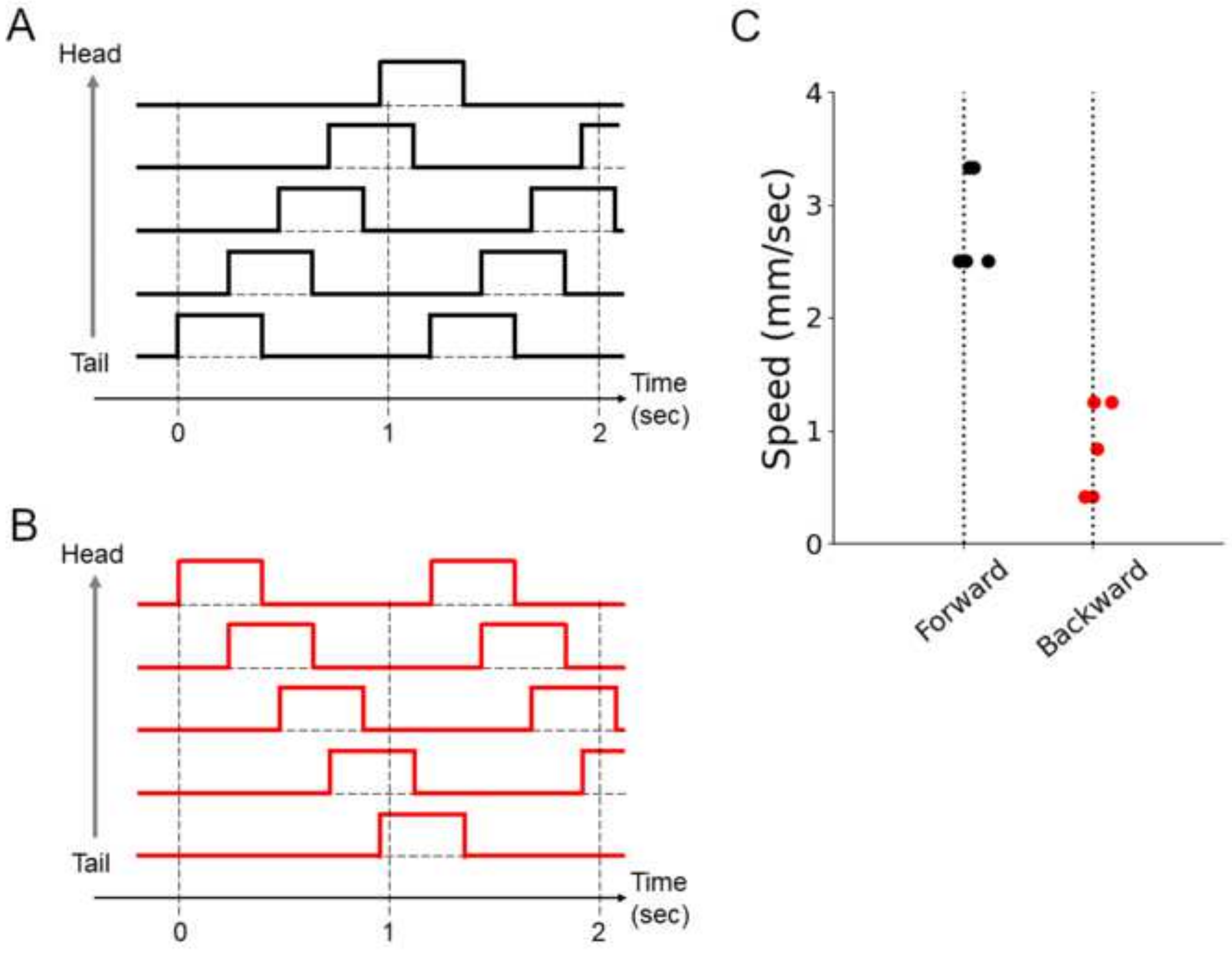

Click here to access/download;Figure;Fig10.tif

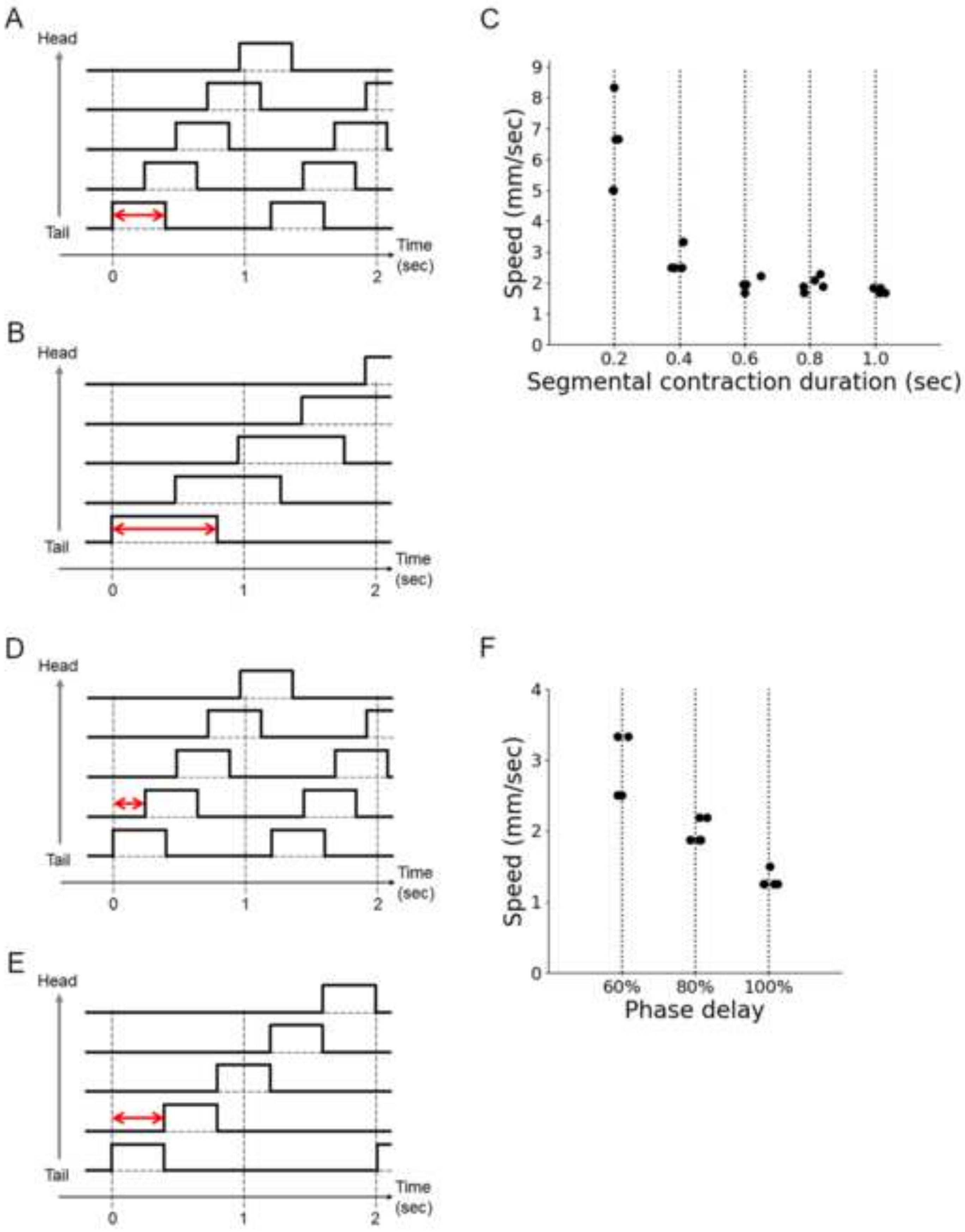

Click here to access/download;Figure;Fig11.tif
Ł æł

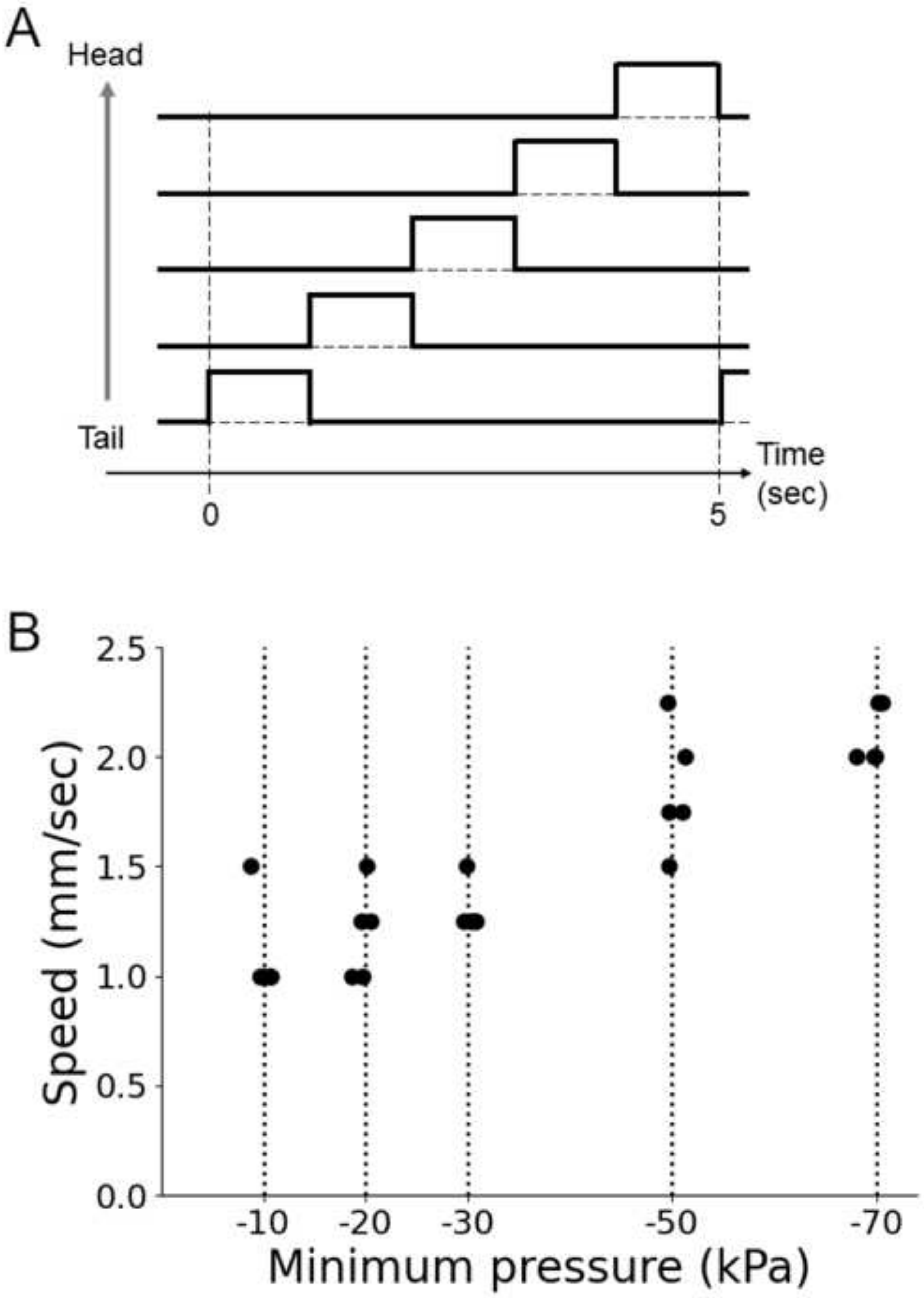

Supplementary Fig1

Click here to access/download
Supporting Information
Supplementary_Fig1.tif

Supplementary Fig2

Click here to access/download
Supporting Information
Supplementary_Fig2.tif

Supplementary Fig3

Click here to access/download
Supporting Information
Supplementary_Fig3.tif

Supplementary Fig4

Click here to access/download
Supporting Information
Supplementary_Fig4.tif
